# Linear and Decoupled Decoders for Dual-Polarized Antenna-Based MIMO Systems

**DOI:** 10.3390/s20247141

**Published:** 2020-12-13

**Authors:** Sara Shakil Qureshi, Sajid Ali, Syed Ali Hassan

**Affiliations:** 1 School of Electrical Engineering & Computer Science (SEECS), National University of Sciences & Technology (NUST), Islamabad 44000, Pakistan; sajid.ali@zu.ac.ae (S.A.); ali.hassan@seecs.edu.pk (S.A.H.); 2 Department of Mathematics and Statistics, College of Natural and Health Sciences, Zayed University, Dubai 19282, UAE

**Keywords:** quaternion orthogonal designs, space time block codes, polarization diversity, decoupled decoding, 5G

## Abstract

Quaternion orthogonal designs (QODs) have been used to design STBCs that provide improved performance in terms of various design parameters. In this paper, we show that all QODs obtained from generic iterative construction techniques based on the Adams-Lax-Phillips approach have linear and decoupled decoders which significantly reduce the computational complexity at the receiver. Our result is based on the quaternionic description of communication channels among dual-polarized antennas. Another contribution of this work is the linear and decoupled decoder for quasi-orthogonal codes for non-square as well as square designs. The proposed solution promises diversity gains with the quaternionic channel model and the decoding solution is independent of the number of receive dual-polarized antennas. A brief comparison is presented at the end to demonstrate the effectiveness of quaternion designs in two dual-polarized antennas over available STBCs for four single-polarized antennas. Linear and decoupled decoding of two quasi-orthogonal designs is shown, which has failed to exit previously. In addition, a QOD for 2×1 dual-polarized antenna configuration using quaternionic channel model shows a 3 dB gain at $10^{-5}$ in comparison to the same code evaluated for 2×2 complex representation of the quaternionic channel. This gain is further enhanced when the received diversity for these the cases is matched i.e., 2×2. The code using the quaternionic channel model shows a further 13 dB improvement at $10^{-5}$ BER.

## 1. Introduction

Wireless communication through multiple antennas is used extensively in today’s telecommunication standards owing to the multiple benefits they offer [1]. More specifically, in addition to providing high data rates through spatial multiplexing, multiple antennas can be used effectively to combat multi-path fading. There are numerous ways in which multiple antennas can be used to provide diversity in wireless signals such as using time, frequency, space, polarization, etc., and the underlying codes carry certain desirable properties such as orthogonality [2]. These properties can be exploited effectively at the receiver side to obtain decoupled solutions with the least complexity. However, because of environmental scattering and imprecise antenna spacing, the diversity gains start to diminish and also pose the problem of coupled solutions, which are computationally expensive at the receiver [3].

With the advent of fifth generation (5G) systems that demand very high data rates and ultra reliability, the paradigm is shifting from simple multiple-input multiple-output (MIMO) to massive MIMO systems where the base station is anticipated to be equipped with hundreds of antennas. Therefore, there is a dire need to study other forms of diversity techniques with new antenna designs that contain, for instance, both polarizations for transmissions and require no extra bandwidth. Polarization diversity enables the simultaneous transmission and reception of information signals using the orthogonally polarized antennas [4]. It has been shown that using a special mathematical tool of quaternion algebra, the transmission/reception/decoding of these MIMO systems can be described effectively [5].

The quaternion orthogonal designs (QODs) are under rigorous research, for instance, see [6,7,8,9,10,11,12]. However, an important construction technique in these previous designs is that they were either extensions of complex orthogonal designs (CODs) with some necessary properties to be applicable as QODs, or they were constructed from quasi-complex space time block codes (STBCs). However, despite the main motivation of exploring polarization diversity in dual-polarized antennas using quaternions, these QODs focused on construction of codes and their decoding.

The decoding of the codes presented previously seem to necessitate that only square orthogonal designs can produce decoupled decoding. In [13], the decoupled decoding solution for quasi-orthogonal codes has been presented based on a wireless communication channel model using the dual-polarized antennas which is derived from the quaternionic channel representation. This model is restricted in terms of its application to only the zero cross polar scattering environments. Also, it restricts the number of dual-polarized antennas at the receiver. Both the above are conservative conditions on a generic wireless communication arrangement. The quaternionic channel proposed in [14] provides codes with optimal rates exploiting polarization diversity along with space and time diversities resulting in higher diversity gains. Dual-polarized antennas are characterized by their polar and cross-polar components. In [14], authors demonstrated the use of quaternionic channel with dual-polarized antennas fully exploits the polarization diversity resulting in higher diversity gains. Also, the use of this system model naturally embeds the cross-polar scattering as demonstrated in [15].

This paper contributes by providing iterative construction techniques for QODs from Adam-Lax-Phillips approach for designing quaternion orthogonal codes using dual-polarized antennas. It is remarkable to note that all generic iterative constructions of QODs result in decoupled and linear decoders with enhanced throughput using the system model proposed in [14] that forms the main result of this paper. Secondly, we identify that there are non-square designs which can have decoupled solutions contrary to what has been believed that these fail to attain decoupled decoding and only pair-wise decoding is possible for them. The proposed design enjoys freedom in exploiting the transmit and receive diversities with no restriction on the antenna dimensions at both the transmitter and receiver ends. It fully exploits the polarization diversity using the cross polar scattering components of the dual-polarized antennas, making it more practical for current and future massive MIMO wireless communication systems. We also briefly compare the performance of QODs for two-input and singe-output (TISO) system of dual-polarized antennas with a 4×2 multiple-input two-output (MITO) system of single-polarized antennas. The former is shown to have key advantages over the latter. In the end, a detailed comparison of the proposed coding and decoding design for the quasi-orthogonal STBCs is evaluated in light of the literature. The main contributions and novelties of this paper include:

- Design of generalized iterative construction techniques for QODs from Adam-Lax-Phillips approach has been proposed.
- For a fully quaternionic channel model, proposal of linear and decoupled decoder for the QODs (i.e., non-square as well as square quasi-orthogonal codes) is presented.
- Seamless extension of the QODs using dual-polarized antennas with freedom of transmit and recieve diversities and antenna dimensions for application to future multiple-input multiple-output (MIMO) systems.

Some remarks on notations are as follows. All matrices are denoted with bold capital letters. *ℜ* and *ℑ* represent the real and imaginary parts of a complex number. The quaternion field Q comprises of a basis of anti-commuting elements 1,i,j,k, such that ij=k=−ji,jk=i=−kj,ki=j=−ik. Quaternion conjugate is denoted with the superscript $q^{Q}$ which changes sign of i,j and *k*. The transpose, Hermititian transpose and trace operators are denoted in standard as ${\left(.\right)}^{T}$, ${\left(.\right)}^{H}$ and tr(.), respectively. Both matrices $C_{q}$ and $C_{Q}$, corresponds to quasi-orthogonal STBCs; however, the subscript *Q* indicates that the STBC $C_{Q}$ is obtained from a QOD.

## 2. Realization of Quaternion Designs

The simultaneous transmission of symbols through a dual-polarized antenna can be modeled through a unified quaternion $q=z_{1}+z_{2}j$, such that symbols $z_{1}$ and $z_{2}$ are transmitted instantaneously through $T_{H}$ (horizontal) and $T_{V}$ (vertical) polarizations, respectively. This line of approach is resurrected in [14] for single-input single-output (SISO) system by implementing an idea that the orthogonal polarization states can be represented as quaternions [16], thereby attaining polarization diversity gain. Unfortunately, for SISO systems the gains from different form of diversities are less apparent and becoms proficient for large number of antennas in MIMO systems. For such systems, it is necessary to develop an iterative approach so that higher order quaternion designs can be generated, which forms the main topic of this paper.

We assume that each quaternion in a QOD comprises of two complex symbols which are obtained from standard modulation schemes, e.g., quadrature phase shift keying (QPSK). To exploit diversity gains from space and time, the orthogonal space-time polarization block codes (OSTPBC) can be defined in the quaternion domain.

**Definition** **1** **(QOD).** A QOD Q, on pure quaternion elements {$q_{1},q_{2},\cdots ,q_{n}$} of type {$s_{1},s_{2},\cdots ,s_{n}$} is an m×n matrix with entries from set { $0,q_{1},q_{1}^{*},q_{2},q_{2}^{*},\cdots ,q_{n},q_{n}^{*}$} including possible multiplications on the left and/or right by quaternion elements q∈Q and satisfying the condition

(1) $$Q^{Q}Q=\sum_{h=1}^{n}\left(s_{h}{\left(\left|q_{h}\right|\right)}^{2}\right)I_{n\times n}=\lambda I_{n\times n}\;,$$

*where $I_{n\times n}$ is the n×n identity matrix and λ is a positive real number.*

In [14], there were three QODs considered (two of order 2×2 and one QOD with order 4×3) which were based on non-iterative construction techniques. In this paper, we demonstrate that higher order QODs can easily be generated iteratively and have fast, linear, and decoupled decoders. Indeed, ref. [3] proposed three generic iterative construction techniques, namely Adams-Lax-Phillips, Józefiak, and Wolfe constructions. A general COD is designed for l+1 symbols embedded in a square matrix of order $2^{l}$ such that

(2) $$A=\left[\begin{array}{cc} G_{2^{l-1}}\left(z_{1},z_{2},\cdots ,z_{l}\right) & z_{l+1}I_{2^{l-1}}\\ -z_{l+1}^{*}I_{2^{l-1}} & G_{2^{l-1}}^{H}\left(z_{1},z_{2},\cdots ,z_{l}\right) \end{array}\right],$$

where l={1,2,3⋯} and $G_{2^{l-1}}\left(z_{1},z_{2},\cdots ,z_{l}\right)$ represents a COD of order $2^{l-1}\times 2^{l-1}$ defined on symbols $\left\{z_{1},z_{2},\cdots ,z_{l}\right\}$. For example, for l=1, $G_{1}\left(z_{1}\right)=\left[z_{1}\right]$.

It is now easy to generate square QODs using above mechanism [17]. We briefly indicate the steps involved in generating a hierarchy of such designs along with the main proof. In particular, by systematically swapping columns $1,2,\cdots ,N_{t}/2$ of matrix A with $\left(N_{t}/2\right)+1,\left(N_{t}/2\right)+2,\cdots ,N_{t}$ columns, respectively, an equivalent matrix B, is generated where $N_{t}$ represents the number of antennas of COD on which permutation is performed. This gives rise to the following result where we have omitted the redundant argument in G for the sake of simplicity.

**Theorem** **1.** *For a given COD A in (2), a complex amicable and symmetric-paired design can be constructed such that the following realization*

$Q_{2^{l}}\left(z_{1},z_{2},..,z_{l+1}\right)=A+Bj=$

(3) $$\left[\begin{array}{cc} G_{2^{l-1}}+z_{l+1}I_{2^{l-1}}j & z_{l+1}I_{2^{l-1}}+G_{2^{l-1}}j\\ -z_{l+1}^{*}I_{2^{l-1}}+G_{2^{l-1}}^{H}j & G_{2^{l-1}}^{H}-z_{l+1}^{*}I_{2^{l-1}}j \end{array}\right],$$

*provides a QOD of dimension $2^{l}\times 2^{l}$ with complex rate $\left(l+1\right)/2^{l}$.*

**Proof.**  To prove the quaternion orthogonality (1), we begin by noticing that $Q_{2^{l}}^{Q}=A^{H}-jB^{H}$, owing to the fact that both A and B are CODs. Hence, $Q_{2^{l}}^{Q}Q_{2^{l}}=\left(A^{H}-jB^{H}\right)\left(A+Bj\right)$, in which the outer product merely yields a Frobenius norm of complex numbers $z_{1},z_{2},\cdots ,z_{l}$ multiplied by an identity matrix. However, the inner product $A^{H}Bj-jB^{H}A$ is identically equal to zero because $A^{H}B=B^{H}A$. This follows from the construction of B which is obtained from A upon permutation of columns. □

For completeness, we delve into another iterative construction technique as it also has decoupled decoding. It is, however, different from the above approach for there is no need for necessarily generating B by permutation.

**Lemma** **1.** *For a given square COD $G_{2^{l-1}}\left(z_{1},z_{2},\cdots ,z_{l+1}\right)$, the matrix*

(4) $$Q_{2^{1}\times 2^{l-1}}\left(z_{1},z_{2},\cdots ,z_{l+1}\right)=\left[\begin{array}{c} G_{2^{l-1}}\left(z_{1},z_{2},\cdots ,z_{l}\right)+z_{l+1}I_{2^{l-1}}j\\ -z_{l+1}^{*}I_{2^{l-1}}+G_{2^{l-1}}^{H}\left(z_{1},z_{2},\cdots ,z_{l}\right)j \end{array}\right]$$

*provides a quaternion design of order $2^{l}\times 2^{l-1}$, with rate $\left(l+1\right)/2^{l}$.*

**Proof.**  The Equation (1) for above code is simplified into $\left(G^{H}+z^{*}I\right)\left(G+zI\right)+\left(-zI+G\right)\left(-z^{*}I+G^{H}\right)$. As before, the outer products of both terms result into Frobenius norm due to the orthogonality of G. The inner product is $G^{H}z+z^{*}G-zG^{H}-Gz^{*}$, which is identically equal to zero due to commutativity of the complex numbers. □

Please note that due to the identity matrix in the term $z_{l+1}I_{2^{l-1}}j$, there will be at least one element in the first time slot which does not contain *j*. Hence, this construction lacks in providing non-zero QODs. Codes with non-zero entries ensure fixed average power codeword by maintaining reduced peak power transmission from every antenna. This results in a favorably low peak-to-average power ratio (PAPR) and diminishes the hardware implications to switch antennas on and off while transmitting a non-zero and zero, respectively [18]. An iterative technique without such a drawback is considered below.

**Lemma** **2.** *For two generalized CODs $G_{2^{l}}\left(z_{1},z_{2},\cdots ,z_{l+1}\right)$ and $L_{2^{l}}\left(z_{l+2},z_{l+3},..,z_{2l+2}\right)$ with same structure, which are constructed on the COD formulation (2), it follows that*

(5) $$\begin{array}{c} G_{2^{l}}^{H}L_{2^{l}}+L_{2^{l}}^{H}G_{2^{l}}=G_{2^{l}}L_{2^{l}}^{H}+L_{2^{l}}G_{2^{l}}^{H}=\gamma I_{2^{l}}, \end{array}$$

where $\gamma =2ℜ\left(\sum_{k=1}^{l+1}z_{k}^{*}z_{l+1+k}\right)$.

It is important to mention that the above lemma does not hold true for two general CODs. For example, two Alamouti codes with different structures $\left[\begin{array}{cc} z_{1} & z_{2}\\ z_{2}^{*} & -z_{1}^{*} \end{array}\right]$ and $\left[\begin{array}{cc} z_{3} & z_{4}\\ -z_{4}^{*} & z_{3}^{*} \end{array}\right]$, fail to satisfy it while they can be used effectively in generating a consistent COD of the form (2). By using the above lemma, we arrive at the following theorem which can be proved in a similar way.

**Theorem** **2.** *For generalized CODs $G_{2^{l-1}}\left(z_{1},z_{2},\cdots .,z_{l}\right)$ and $L_{2^{l-1}}\left(z_{1+2},z_{2},\cdots ,z_{2l+2}\right)$, a symmetric-paired design,*

(6) $$Q_{2^{l+1}\times 2^{l}}\left(z_{1},\cdots ,z_{2(l+1)}\right)=\left[\begin{array}{c} G_{2^{l}}+L_{2^{l}}j\;,\\ L_{2^{l}}+G_{2^{l}}j \end{array}\right],$$

*is a QOD of dimension $2^{l+1}\times 2^{l}$ with complex rate $\left(l+1\right)/2^{l}$.*

QODs are evaluated for the proposed construction technique for (2×1), (4×1) and (8×1) dual-polarized antenna arrangements in subsequent sections.

## 3. Higher Order Designs for Dual-Polarized Antennas

### 3.1. Designs for (2 × 1)-Dual-Polarized Antennas

To elaborate the generalized construction technique, we present a QOD of rate 1, where the COD A contains symbols $z_{1}$ and $z_{2}$, while the COD B contains independent symbols $z_{3}$ and $z_{4}$, respectively. Using (3), we obtain the following symmetric-paired design of order 4×2 with a complex code rate of 1,

(7) $$\begin{array}{c} Q_{1}=\left[\begin{array}{cc} z_{1}+z_{3}j & z_{2}+z_{4}j\\ z_{2}^{*}+z_{4}^{*}j & -z_{1}^{*}-z_{3}^{*}j\\ z_{3}+z_{1}j & z_{4}+z_{2}j\\ z_{4}^{*}+z_{2}^{*}j & -z_{3}^{*}-z_{1}^{*}j \end{array}\right], \end{array}$$

where l=1, $G_{1}=\left[z_{1}\right]$, and $L_{1}=\left[z_{3}\right]$ from (3). Please note that in the above code, both ends of a dual-polarized antenna will be used at each time slot. Therefore, the QODs obtained through this procedure will contain non-zero complex symbols in each time slot.

We consider the following example for illustration. Unlike [17], we will use this design in our proposed system model and show that it has decoupled decoding without the need for applying projection operator.

#### Distinctiveness of QODs

An interesting property of QODs which distinguishes them from CODs is that there exists QODs of complex rate greater than one, which have decoupled decoders. For instance, the following QOD has code rate 2 and is shown to posses decoupled decoder

(8) $$Q_{2}=\left[\begin{array}{cc} z_{1}+z_{2}j & z_{4}+z_{3}j\\ z_{2}^{*}-z_{1}^{*}j & -z_{3}^{*}+z_{4}^{*}j \end{array}\right].$$

The main reason behind is that the Alamouti code is proposed for single-polarized antennas while QODs are developed for dual-polarized antennas. Similarly, there is a QOD of rate 1 which is given as

(9) $$Q_{3}=\left[\begin{array}{cc} z_{1}+z_{2}j & j(z_{1}+z_{2}j)\\ i(z_{1}+z_{2}j) & -k(z_{1}+z_{2}j), \end{array}\right].$$

which provides decoupled decoding.

### 3.2. Design for (4 × 1)-Dual-Polarized Antennas

We start with an Alamouti code $G_{2}=\left[\begin{array}{cc} z_{1} & z_{2}\\ -z_{2}^{*} & z_{1}^{*} \end{array}\right]$, then using (2) we first obtain a COD of order 4. Through permutations, the matrix B is generated using (6) and finally we get the following QOD for 4 dual-polarized antennas with rate 3/4

$$Q_{4}=\left[\begin{array}{cccc} z_{1}+z_{3}j & z_{2} & z_{3}+z_{1}j & z_{2}j\\ -z_{2}^{*} & z_{1}^{*}+z_{3}j & -z_{2}^{*}j & z_{3}+z_{1}^{*}j\\ -z_{3}^{*}+z_{1}^{*}j & -z_{2}j & z_{1}^{*}-z_{3}^{*}j & -z_{2}\\ z_{2}^{*}j & -z_{3}^{*}+z_{1}j & z_{2}^{*} & z_{1}-z_{3}^{*}j \end{array}\right].$$

In comparison to the last three QODs, the above QOD suffers one drawback that in each time slot we have to switch-off two polarizations (*H* or *V*) of at least two dual-polarized antennas, which results in a high peak-to-average power ratio and is not practically desirable.

### 3.3. Design for (8 × 1)-Dual-Polarized Antennas

We start with a maximal rate square COD of order $2^{2}=4$ given by

(10) $$G_{4}=\left[\begin{array}{cccc} z_{1} & z_{2} & z_{3} & 0\\ z_{2}^{*} & -z_{1}^{*} & 0 & z_{3}\\ z_{3}^{*} & 0 & z_{1}^{*} & z_{2}\\ 0 & z_{3}^{*} & z_{2}^{*} & -z_{1} \end{array}\right],$$

as a seed matrix to generate the required QOD using (6). Thus, for a massive MIMO comprising of an 8×1 system, we have a QOD which has code rate 1/2 and is given in Equation (11).

(11) $$Q_{5}=\left[\begin{array}{cccccccc} z_{1}+z_{4}j & z_{2} & z_{3} & 0 & z_{4}+z_{1}j & z_{2}j & z_{3}j & 0\\ z_{2}^{*} & -z_{1}^{*}+z_{4}j & 0 & z_{3} & z_{2}^{*}j & z_{4}-z_{1}^{*}j & 0 & z_{3}j\\ z_{3}^{*} & 0 & z_{1}^{*}+z_{4}j & z_{2} & z_{3}^{*}j & 0 & z_{4}+z_{1}j & z_{2}j\\ 0 & z_{3}^{*} & z_{2}^{*} & -z_{1}+z_{4}j & 0 & z_{3}^{*}j & z_{2}^{*}j & z_{4}-z_{1}j\\ -z_{4}^{*}+z_{1}^{*}j & z_{2}j & z_{3}j & 0 & z_{1}^{*}-z_{4}^{*}j & z_{2} & z_{3} & 0\\ z_{2}^{*}j & -z_{4}^{*}-z_{1}j & 0 & z_{3}j & z_{2}^{*} & -z_{1}-z_{4}^{*}j & 0 & z_{3}\\ z_{3}^{*}j & 0 & -z_{4}^{*}+z_{1}j & z_{2}j & z_{3}^{*} & 0 & z_{1}-z_{4}^{*}j & z_{2}\\ 0 & z_{3}^{*}j & z_{2}^{*}j & -z_{4}^{*}-z_{1}^{*}j & 0 & z_{3}^{*} & z_{2}^{*} & -z_{1}^{*}-z_{4}^{*}j \end{array}\right].$$

## 4. System Model and Decoding

**Figure 1:**
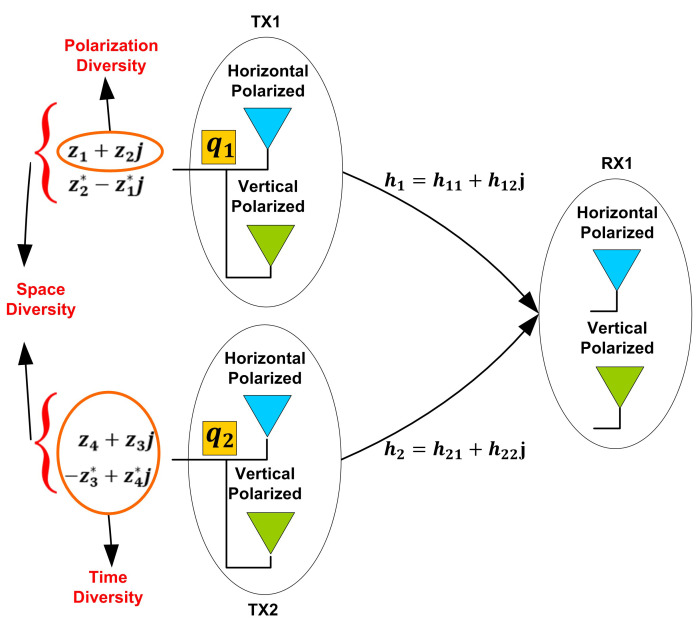
Two-input single-output (TISO) dual-polarized antenna configuration exploiting space, time and polarization diversities.

We first consider a two-input and single-output system (TISO) of dual-polarized antennas where it is necessary to emphasize the role of quaternions which is more recognizable in this case, therefore, we have

(12) $$R=\left[\begin{array}{c} r_{1}\\ r_{2} \end{array}\right]=\left[\begin{array}{cc} q_{1} & q_{2}\\ q_{3} & q_{4} \end{array}\right]\left[\begin{array}{c} h_{1}\\ h_{2} \end{array}\right]+\left[\begin{array}{c} n_{1}\\ n_{2} \end{array}\right],$$

where each element in the above construction is a quaternion. Through first antenna in the above TISO system, the transmission of a pair of two complex symbols is encoded in $q_{1}$ and another pair in $q_{3}$. This indicates that the above QOD exploits time and space diversities along with polarization diversity, as shown in Figure 1. It is worth pointing out that each quaternionic product, e.g., $q_{a}h_{b}$ contains a crucial information about the nature of quaternion domain. If we decompose it for a general quaternionic product then we obtain $q_{a1}h_{b1}-q_{a2}h_{b2}+j\left(q_{a1}h_{b2}+q_{a2}h_{b1}\right)$, where $q_{a}=q_{a1}+jq_{a2}$ and $h_{b}=h_{b1}+jh_{b2}$. Therefore, we obtain four complex channel gains for each antenna in a 2×1 system. Subsequently, a system model for a MISO system of dual-polarized antennas can be constructed in the same way for such a system with $N_{T}\times 1$ dual-polarized antennas

(13) $$R_{T\times 1}=Q_{T\times N_{T}}H_{N_{T}\times 1}+N_{T\times 1},$$

which transmits symbols in T−times slots where $H=[h_{1},h_{2},\cdots ,h_{N_{T}}]$, such that each entry is a quaternion $h_{a}=h_{a1}+h_{a2}j,$ for all $a\in \{1,2,\cdots ,N_{T}\}$. The complex channel gains, $h_{a1}$ and $h_{a2}$ incorporate the effects of cross polar scattering and the channel is assumed to be Rayleigh fading, which implies that each element of channel gain matrix is a complex Gaussian random variable (RV) with zero mean and unit variance. Moreover, the noise $N=[n_{1},n_{2},\cdots ,n_{T}]$, and $n_{b}=n_{b1}+n_{b2}j$, such that $n_{b1},n_{b2}\;\forall \;b=\left\{1,2,\cdots ,T\right\}$, represent the entries of white noise as two dimensional independent and identically distributed (i.i.d.) complex Gaussian RVs with zero mean and identical variance per dimension.

Based on the system model given in (13), the following theorem confirms a linear decoupled solution at the receiver for all QODs constructed in the previous section.

**Theorem** **3.** *For a given system model in (13), the ML-decoding rule assumes a linear decoupled form*

(14) $$\begin{array}{cc} \min_{z}{\left||R-QH|\right|}^{2}= & \min_{z}(tr\left(R^{Q}R\right)+\lambda tr\left(H^{Q}H\right)-\\ \; & 2ℜ\left(tr\left(R^{Q}QH\right)\right)). \end{array}$$

Therefore, the ML-decoding rule reduces to minimizing $ℜ\left(tr\left(R^{Q}QH\right)\right)$, because the first two terms in (14) are constants. There are two main advantages of the above ML-decoding rule. The presence of QOD, Q, in the term $ℜ\left(tr\left(R^{Q}QH\right)\right)$, contributes only linear terms of complex symbols. Secondly, it significantly reduces the computational load at the receiver for the reason that the term $ℜ\left(tr\left(R^{Q}QH\right)\right)$, can easily be expressed without involving matrices at all which may be cumbersome for large MIMO systems.

It is emphasized here that for all QODs obtained in the previous section, the decoupled decoding rule, similar to Corollary 1, can be derived explicitly. As an illustration of the above result, we choose among them the QODs given in (7) and (8) and demonstrate that the above ML-decoding rule is both linear and decoupled.

**Corollary** **1.** *The ML-decoding rule (14) for QOD given in (7), reduces to*

(15) $$\begin{array}{cc} \; & -2\min_{z_{1}}ℜ\left(r_{1}^{q}z_{1}h_{1}-r_{2}^{q}z_{1}^{*}h_{2}+r_{3}^{q}z_{1}h_{1}j-r_{4}^{q}z_{1}^{*}h_{2}^{*}j\right)\;,\\ \; & -2\min_{z_{2}}ℜ\left(r_{1}^{q}z_{2}h_{2}+r_{2}^{q}z_{2}^{*}h_{1}+r_{3}^{q}z_{2}h_{2}^{*}j+r_{4}^{q}z_{2}^{*}h_{1}^{*}j\right)\;,\\ \; & -2\min_{z_{3}}ℜ\left(r_{1}^{q}z_{3}h_{1}^{*}j-r_{2}^{q}z_{3}^{*}h_{2}^{*}j+r_{3}^{q}z_{3}h_{1}-r_{4}^{q}z_{3}^{*}h_{2}\right)\;,\\ \; & -2\min_{z_{4}}ℜ\left(r_{1}^{q}z_{4}h_{2}^{*}j+r_{2}^{q}z_{4}^{*}h_{1}^{*}j+r_{3}^{q}z_{4}h_{2}+r_{4}^{q}z_{4}^{*}h_{1}\right)\;, \end{array}$$

*where $R={\left[r_{1}\;r_{2}\;r_{3}\;r_{4}\right]}^{T},$ is the received vector with each element is a quaternion and $h_{1}=h_{11}+h_{12}j$ and $h_{2}=h_{21}+h_{22}j.$*

The ML-decoding rule (14) for QOD given in (11), reduces to

(16) $$\begin{array}{cc} & -2\min_{z_{1}}ℜ(r_{1}^{q}z_{1}h_{1}+r_{1}^{q}z_{1}h_{5}^{*}j-r_{2}^{q}z_{1}^{*}h_{2}-r_{2}^{q}z_{1}^{*}h_{6}^{*}j+r_{3}^{q}z_{1}^{*}h_{3}-r_{3}^{q}z_{1}^{*}h_{7}^{*}j-r_{4}^{q}z_{1}h_{4}-r_{4}^{q}z_{1}h_{3}^{*}j+r_{5}^{q}z_{1}^{*}h_{1}^{*}j+r_{5}^{q}z_{1}^{*}h_{5}\\ & -r_{6}^{q}z_{1}h_{2}^{*}j-r_{6}^{q}z_{1}h_{6}+r_{7}^{q}z_{1}h_{3}^{*}j+r_{7}^{q}z_{1}h_{7}-r_{8}^{q}z_{1}^{*}h_{4}^{*}j-r_{8}^{q}z_{1}^{*}h_{8})\;,\\ & -2\min_{z_{2}}ℜ(r_{1}^{q}z_{2}h_{2}+r_{1}^{q}z_{2}h_{6}^{*}j+r_{2}^{q}z_{2}^{*}h_{1}+r_{2}^{q}z_{2}^{*}h_{5}^{*}j+r_{3}^{q}z_{2}h_{4}+r_{3}^{q}z_{2}h_{8}^{*}j+r_{4}^{q}z_{2}^{*}h_{3}+r_{4}^{q}z_{2}^{*}h_{7}^{*}j+r_{5}^{q}z_{2}h_{2}^{*}j+r_{5}^{q}z_{2}h_{6}\\ & +r_{6}^{q}z_{2}^{*}h_{1}^{*}j+r_{6}^{q}z_{2}^{*}h_{5}+r_{7}^{q}z_{2}h_{4}^{*}j+r_{7}^{q}z_{2}h_{8}+r_{8}^{q}z_{2}^{*}h_{3}^{*}j+r_{8}^{q}z_{2}^{*}h_{7})\;,\\ & -2\min_{z_{3}}ℜ(r_{1}^{q}z_{3}h_{3}+r_{1}^{q}z_{3}h_{7}^{*}j+r_{2}^{q}z_{3}h_{4}+r_{2}^{q}z_{3}h_{8}^{*}j+r_{3}^{q}z_{3}^{*}h_{1}+r_{3}^{q}z_{3}^{*}h_{5}^{*}j+r_{4}^{q}z_{3}^{*}h_{2}+r_{4}^{q}z_{3}^{*}h_{6}^{*}j+r_{5}^{q}z_{3}h_{3}^{*}j+r_{5}^{q}z_{3}h_{7}\\ & +r_{6}^{q}z_{3}h_{4}^{*}j+r_{6}^{q}z_{3}h_{8}+r_{7}^{q}z_{3}^{*}h_{1}^{*}j+r_{7}^{q}z_{3}^{*}h_{5}+r_{8}^{q}z_{3}^{*}h_{2}^{*}j+r_{8}^{q}z_{3}^{*}h_{6})\;,\\ & -2\min_{z_{4}}ℜ(r_{1}^{q}z_{4}h_{1}^{*}j+r_{1}^{q}z_{4}h_{5}+r_{2}^{q}z_{4}h_{2}^{*}j+r_{2}^{q}z_{4}h_{6}+r_{3}^{q}z_{4}h_{3}^{*}j+r_{3}^{q}z_{4}h_{7}+r_{4}^{q}z_{4}h_{4}^{*}j+r_{4}^{q}z_{4}h_{8}-r_{5}^{q}z_{4}^{*}h_{1}-r_{5}^{q}z_{4}^{*}h_{5}^{*}j\\ & -r_{6}^{q}z_{4}^{*}h_{2}-r_{6}^{q}z_{4}^{*}h_{6}^{*}j-r_{7}^{q}z_{4}^{*}h_{3}-r_{7}^{q}z_{4}^{*}h_{7}^{*}j-r_{8}^{q}z_{4}^{*}h_{4}-r_{8}^{q}z_{4}^{*}h_{8}^{*}j)\;, \end{array}$$

Please note that we have four complex channel gains between a TISO system of dual-polarized antennas, as shown in Figure 1. As this system is equivalent to a MIMO 4×2 system of single-polarized antennas, therefore, it may appear that it should have eight channel gains in total with two for each link. However, in our proposed model each quaternionic product results in the same number of channel gains. The receiver now computes the decision metric $\min_{z}{\left||R-QH|\right|}^{2}$, which involves matrices. On the other hand, an optimal decoder (14) is also used to receive signal that significantly reduces total time consumed. In the end, we give the decoder for QOD (11).

**Corollary** **2.** *The ML-decoding rule (14) for QOD (11) is given in (16) where $R={\left[r_{1}\;r_{2}\;r_{3}\;r_{4}\;r_{5}\;r_{6}\;r_{7}\;r_{8}\right]}^{T},$ is the received vector with each element is a quaternion and $h_{i}=h_{i1}+h_{i2}j,i=1,2,...,8$.*

## 5. Key Aspects of QODs under Quaternion Channel

### 5.1. Comparison with Benchmark Codes

**Figure 2:**
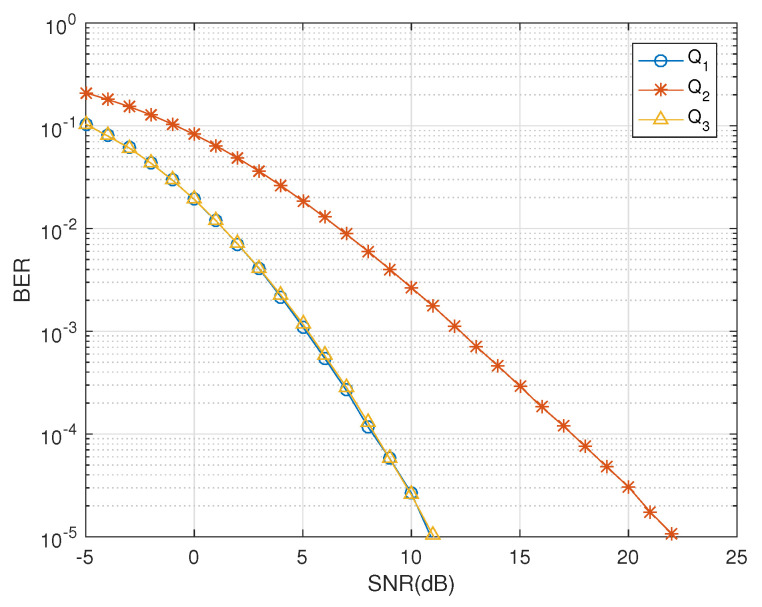
BER vs. SNR performance of QODs $Q_{1},Q_{2}$ and $Q_{3}$.

**Figure 3:**
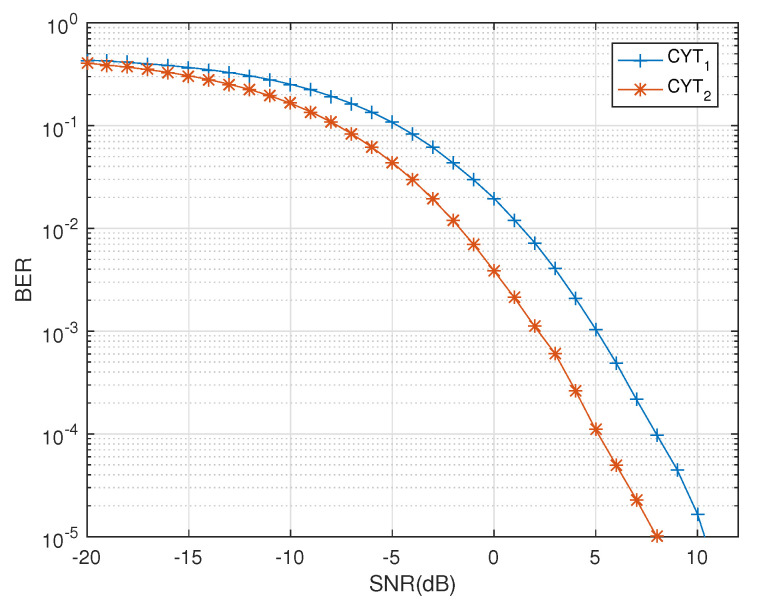
BER vs. SNR performance of QODs ${\mathrm{CYT}}_{1}$ and ${\mathrm{CYT}}_{2}$.

Dual-polarized antennas can exploit the space, time and polarization diversities suitably and the designed codes based on QODs are used to serve this purpose. The BER performance of the codes $Q_{1},Q_{2}$ and $Q_{3}$ is given in Figure 2. Notice that the designs $Q_{1}$ and $Q_{3}$ have overlapping BER curves, attaining the same diversity gains; however, $Q_{3}$ has a relatively better throughput than $Q_{1}$. We now perform a consolidated comparison of QODs developed for two dual-polarized antennas against conventional STBCs designed for four single-polarized antennas to indicate the major differences. For four transmit single-polarized antennas, the authors in [19,20] used *amicable designs* to construct minimum decoding quasi STBCs that essentially require the products $A^{H}B$ and $B^{H}A$ to be equal where A and B are amicable STBCs. This drastically reduces the code rate which in our designs remain stable as they require only the property of being symmetric property. In particular, for four and eight transmit antennas, there are two “square” STBCs constructed (Equation (20) in [19]), which have rates 1 and 3/4, respectively. We denote their STBCs with **CYT** given as

(17) $${\mathrm{CYT}}_{1}=\left[\begin{array}{cccc} z_{1} & z_{2} & -z_{3} & -z_{4}\\ -z_{2}^{*} & z_{1}^{*} & -z_{4}^{*} & z_{3}^{*}\\ z_{3} & z_{4} & z_{1} & z_{2}\\ z_{4}^{*} & -z_{3}^{*} & -z_{2}^{*} & z_{1}^{*} \end{array}\right],$$

where $z_{1}=c_{2}^{R}+jc_{3}^{I}$, $z_{2}=c_{2}^{R}+jc_{4}^{I}$, $z_{3}=c_{3}^{R}+jc_{1}^{I}$ and $z_{4}=c_{4}^{R}+jc_{1}^{I}$, where *j* refers to imaginary unit. Previously, the authors in [20] obtained the following square OSTBC (Equation (11) in [20])

(18) $${\mathrm{CYT}}_{2}=\left[\begin{array}{cccc} x_{1}^{*}-x_{2} & x_{1}^{*}+x_{2} & x_{3}^{*} & -x_{3}^{*}\\ jx_{1}+jx_{2}^{*} & -jx_{1}+jx_{2}^{*} & jx_{3}^{*} & jx_{3}^{*}\\ -x_{3} & x_{3} & x_{1}^{*}-x_{2}^{*} & x_{1}^{*}+x_{2}^{*}\\ -jx_{3} & -jx_{3} & jx_{1}+jx_{2} & -jx_{1}+jx_{2} \end{array}\right],$$

which was shown to have significant performance edges over previously known codes proposed in [21,22]. Because of the orthogonality condition, the above code ${\mathrm{CYT}}_{2}$ has less code rate than quasi code ${\mathrm{CYT}}_{1}$. Figure 3 provides a comparison of the quaternion equivalent of the codes ${\mathrm{CYT}}_{1}$ and ${\mathrm{CYT}}_{2}$ using the channel model presented in [14]. These codes have complex receivers as they fail to have decoupled decoders in complex domain. In this work, linear and decoupled decoding of these codes is possible due to the proposed decoder design based on the channel model presented in [14]. On the other hand, the state-of-art linear dispersion STBCs are proposed for four transmit antennas in [23] of maximal rate 1 when the distance between transmit antennas satisfies a physical constraint.

For a brief fair comparison of QODs with benchmark codes, we construct the complex analogues of QODs by applying operator C such that $C\left(z_{1}+z_{2}j\right)=\left[z_{1}\;z_{2}\right]$. In this way, we obtain four equivalent quasi-codes (yet quaternion orthogonal) for four transmit single-polarized antennas given as

(19) $$\begin{array}{cc} \; & C_{Q_{1}}=\left[\begin{array}{cccc} z_{1} & z_{3} & z_{2} & z_{4}\\ z_{2}^{*} & z_{4}^{*} & -z_{1}^{*} & -z_{3}^{*}\\ z_{3} & z_{1} & z_{4} & z_{2}\\ z_{4}^{*} & z_{2}^{*} & -z_{3}^{*} & -z_{1}^{*} \end{array}\right], \end{array}$$

(20) $$\begin{array}{cc} \; & C_{Q_{2}}=\left[\begin{array}{cccc} x_{3}+x_{4}i & x_{0}+x_{2}i & x_{3}+x_{4}i & x_{1}+x_{2}i\\ x_{3}+x_{4}i & -x_{1}+x_{2}i & -x_{3}-x_{4}i & x_{0}-x_{2}i \end{array}\right], \end{array}$$

(21) $$\begin{array}{cc} \; & C_{Q_{3}}=\left[\begin{array}{cccc} z_{1} & z_{2} & z_{3} & z_{4}\\ z_{2}^{*} & -z_{1}^{*} & -z_{4}^{*} & z_{3}^{*} \end{array}\right], \end{array}$$

(22) $$\begin{array}{cc} \; & C_{Q_{4}}=\left[\begin{array}{cccc} z_{1} & z_{2} & z_{1}^{*} & -z_{2}^{*}\\ iz_{1} & iz_{2} & iz_{2}^{*} & -iz_{1}^{*} \end{array}\right], \end{array}$$

with which a comprehensive comparison can be done with ${\mathrm{CYT}}_{1}$ or ${\mathrm{CYT}}_{2}$. Subsequently, we carry out a detailed comparative analysis, which proves that the designs developed in the quaternion domain have performance edge at many fronts such as computational complexity, improved throughput, exploitation of polarization diversity, decoding delays and linear decoupled decoding, etc.

### 5.2. Computational Complexity

The computational complexity in the proposed decoupled decoder is $O\left(4\left(N\right)\left(T\right)2\right)$, where *N* is the number of transmit antennas and *T* is the number of timeslots used to transmit one code block. For the coupled case, this computational complexity has been dependent on ζ and has been defined as $O\left(4^{\zeta }\left(N\right)\left(T\right)2\right)$. The computational complexity of the proposed decoder is dependent only on *N* and *T*. This results in promising gains in terms of the computational complexity of the proposed decoupled decoder in comparison to the coupled decoder. Detailed complexity analysis of the proposed decoupled decoder for $Q_{1}$–$Q_{5}$ is presented in Table 1.

**Figure 4:**
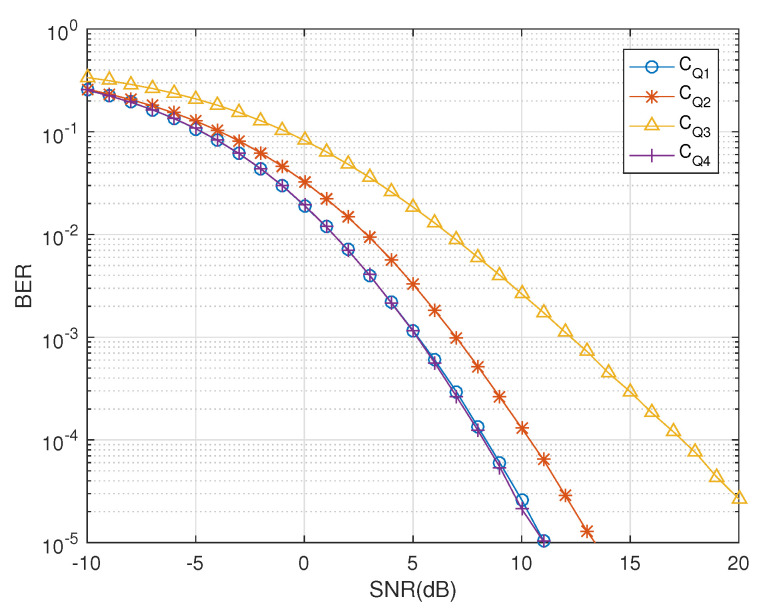
BER vs. SNR performance of QODs $C_{Q_{1}},C_{Q_{2}},C_{Q_{3}}$ and $C_{Q_{4}}$.

Table 1 shows the computational complexity for the QODs. Additionally, the complexity of the receiver for the quasi-orthogonal codes, e.g., $C_{Q1}$, $C_{Q2}$, $C_{Q3}$ and $C_{Q4}$, has also been addressed as the proposed solution provides a decoupled decoding solution using the quaternionic channel, as shown in Figure 4, where the coupled ML decoder would have failed completely.

In Table 2, we briefly summarize the main points which play a dominant role in the quaternion domain when employed for dual-polarized antennas.

### 5.3. Number of Receive Antennas

**Figure 5:**
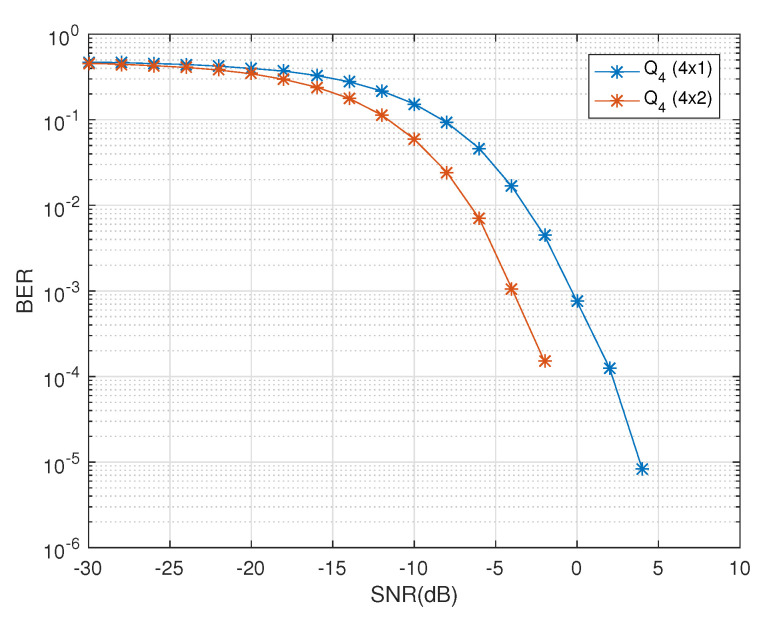
BER Vs SNR performance of $Q_{4}$ for (4×1) and (4×2) antenna configurations.

The physical implementation of the design in [13] is limited with the use of even number of dual-polarized antennas at the receiver. For massive MIMO systems, this is space and cost inefficient with restrictions on the freedom of diversity at the receiver end. The proposed model works for any number of receive dual-polarized antennas, $N_{R}$, i.e., ($N_{T}\times 1),\cdots ,(N_{T}\times N_{R}$); $N_{R}\geq 1$. In Figure 5, the diversity gains for QOD $Q_{4}$ due to the increase in number of receive dual-polarized antennas has been demonstrated.

### 5.4. Diversity Gain

**Figure 6:**
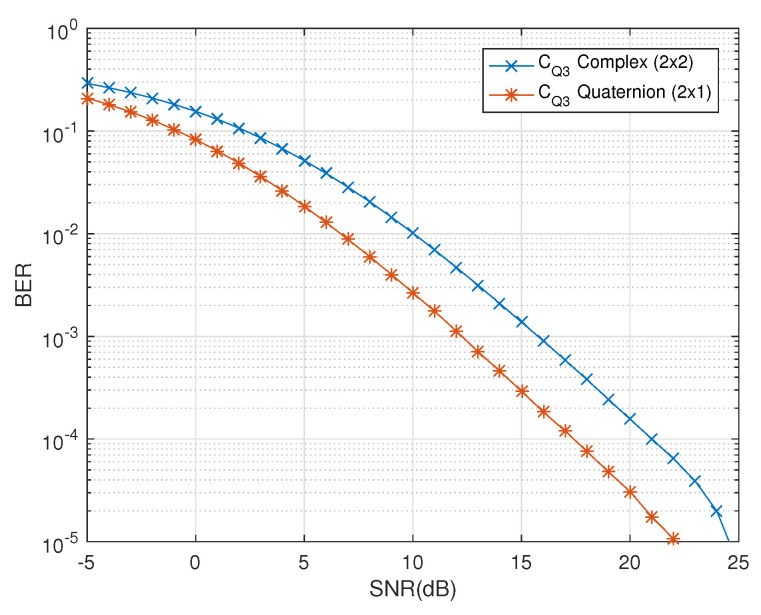
BER vs. SNR performance comparison of $C_{Q_{3}}$ for decoupled decoder in [13] and the quaternionic channel-based decoder.

Diversity is influenced by the change in number of receive antennas. In [13], the authors addressed the problem of decoding in quasi-orthogonal codes. The complex representation of the quaternionic channel was considered with zero cross-polar scattering and constraints on the number of receive dual-polarized antennas. This solution had limited benefits due to its reservations in independently exploiting the polarization diversity with the combination of quaternions and dual-polarized antennas. In contrast, the proposed solution provides the decoupled decoding of non-square as well as square quasi-orthogonal codes without any restrictions on the environment as well as the antenna dimensions. In Figure 6, the effects of restricting the cross-polar scattering has been highlighted. It is evident that ‘$C_{Q_{3}}$ Quaternion 2×1’ shows a diversity gain of about 3 dB at the BER of $10^{-5}$ in comparison to the ‘$C_{Q_{3}}$ Complex 2×2’. This clearly shows that the quaternionic channel model fully exploits the polarization diversity using the dual-polarized antennas with the effects of cross-polar scattering naturally embedded into the quaternion representation of channel. Thus, the use of quaternionic channel model provides diversity gains in comparison to its complex channel equivalents as well as the complex representation of the quaternionic channel model [13].

**Figure 7:**
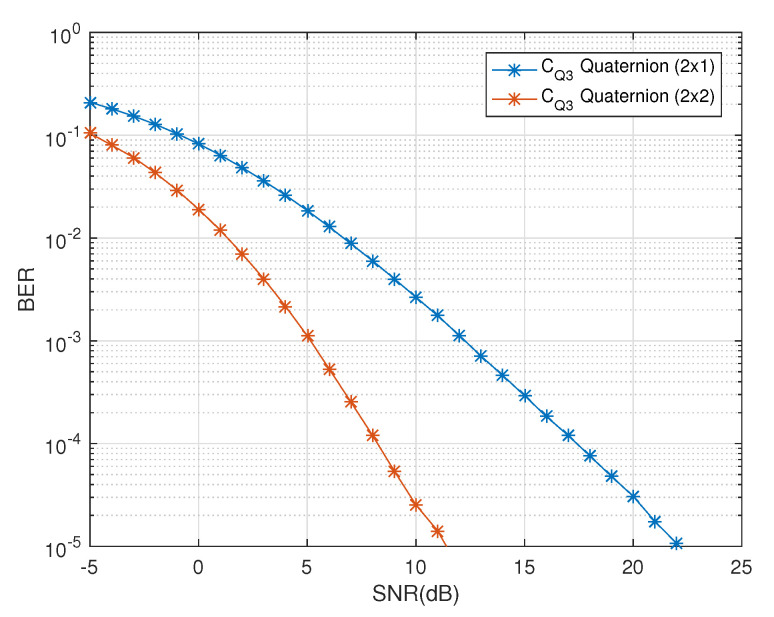
BER vs. SNR performance of $C_{Q_{3}}$ for one and two receive dual-polarized antennas.

In Figure 7, the effect of the freedom of the receive diversity is shown. A decoupled decoding solution of quasi-orthogonal codes has been presented here without any restriction on the cross-polar scattering and the number of receive antennas. In comparison, in [13], the authors presented a decoupled decoding solution using the complex representation of the quaternionic channel model under the constraint conditions of zero cross-polar scattering and number of receive dual-polarized antennas. In Figure 6, ‘$C_{Q_{3}}$ Quaternion 2×1’ with one receive dual-polarized antenna shows better performance in comparison to ‘$C_{Q_{3}}$ Complex 2×2’ with two receive dual-polarized antennas. If the receiver antenna dimensions of these two codes (i.e., ‘$C_{Q_{3}}$ Quaternion’ and ‘$C_{Q_{3}}$ Complex’) are matched (i.e., (2×2)), we can see the diversity gain of ‘$C_{Q_{3}}$ Quaternion 2×2’, in Figure 7, is about 13 dB at BER of $10^{-5}$ when compared with ‘$C_{Q_{3}}$ Complex 2×2’, in Figure 6. Thus, we can see that the freedom in receiver antenna dimensions has a huge impact on the diversity gain when quaternionic channel model is used with the dual-polarized antennas. Also, the best exploitation of polarization diversity is executed using the polar as well as the cross-polar components without any dependence on the number of receive antennas.

### 5.5. Cross-Polar Scattering

Scattering and reflections result in polarization variations where cross polar scattering is natural. The orthogonal quaternion codes are decomposed into quasi-orthogonal STBCS in [13] to provide a decoupled decoding solution. Yet, this model has constraints to have zero cross polar scattering environment, a limiting scenario in real communication systems. Such an exercise appears redundant as decoupled decoding solution for dual-polarized antennas based on a generalized quaternionic channel model was already detailed in [14], which considers both the polar as well as non-cross polar scattering and provides linear decoupled decoding solution for quasi-orthogonal STBCs.

This work presents a generalized decoupled decoding solution for the quasi-orthogonal STBCs using the quaternionic channel model irrespective of any constraints regarding the cross polar scattering, the number of received dual-polarized antennas and coding/decoding delays. This design provides a decoupled decoding solution for any number of transmit and receive dual-polarized antennas.

## 6. Conclusions

This paper presented an evaluation of the conditions employed on the construction of QODs that achieve better diversity gains by exploiting space, time and polarization diversities using quaternion algebra. Iterative construction techniques for QODs have been presented based on Adam-Lax-Phillips approach for dual-polarized antennas. A remarkable contribution of this work is linear and decoupled decoding solution of codes including square as well as non-square quasi-orthogonal designs, which failed in the past. Additionally, the solution presented here is generalized for both polar as well as cross polar scattering environments and is independent of the number of receive dual-polarized antennas. The diversity gains due to the proposed solutions for coding and decoding of QODs are promising. In future, evaluation of this proposal for generating non-zero codes will be interesting. Evaluation of these iterative construction techniques for higher dimensions is another direction to explore. This work can also be validated in future using practical testing to compare the theoretical diversity gains to the findings of the real channel models between the single and dual-polarized antennas with similar antennas dimensions.

## Figures and Tables

**Table 1:** Comparison between the computational complexity of the proposed decoupled decoder.

(ζ, N, T)	(4,4,4)	(4,4,2)	(2,4,2)	(3,8,4)	(4,16,8)
Coupled decoder	8192	4096	256	4096	65,536
Decoupled decoder	128	64	64	256	1024
Percentage improvement	98.44%	98.44%	75%	93.75%	98.44%

**Table 2:** Significant features of the quaternion domain and their comparison with their counter codes in complex domain.

	Complex Designs		Quaternion Designs			
	${\mathrm{CYT}}_{1}$	${\mathrm{CYT}}_{2}$	$Q_{1}$	$Q_{2}$	$Q_{3}$	$Q_{4}$
Type	Quasi	Orthogonal	Orthogonal	Orthogonal	Orthogonal	Orthogonal
Code Rates	1	3/4	1	2	1	3/4
Coding/Decoding Delay	✓	✓	×	×	×	×
Decoupled Decoder	×	×	✓	✓	✓	✓
Space & Time Diversities	✓	✓	✓	✓	✓	✓
Polarization Diversity	×	×	✓	✓	✓	✓

## Abbreviations

The following abbreviations are used in this manuscript:

H	Horizontal
V	Vertical
STBC	Space Time Block Codes
COD	Complex Orthogonal Designs
QOD	Quaternion Orthogonal Designs
5G	Fifth Generation
MIMO	Multiple-Input Multiple-Output
SISO	Single-Input Single-Output
TISO	Two-Input Single-Output
MISO	Multiple-Input Single-Output
OSTPBC	Orthogonal Space Time Polarization Block Code
QPSK	Quadrature Phase Shift Keying
PAPR	Peak-to-Average Power Ratio
RV	Random Variable
FLOPs	Floating Point Operations
BER	Bit Error Rate
SNR	Signal-to-Noise Ratio
